# Role of central and peripheral serotonin in liver physiology and diseases

**DOI:** 10.7150/thno.126199

**Published:** 2026-01-01

**Authors:** Arnauld Belmer, Carmelo Luci, Philippe Gual

**Affiliations:** Université Côte D'Azur, INSERM, U1065, C3M, Nice, France.

## Abstract

Steatotic liver diseases (SLD) associated with metabolic dysfunction (Metabolic dysfunction-Associated Steatotic Liver Disease: MASLD), chronic alcohol consumption (alcohol-associated liver disease: ALD), or both (MetALD) represent a major health issue worldwide. These chronic liver diseases are major drivers of fibrosis, cirrhosis, hepatocellular carcinoma (HCC), and liver-related mortality, with limited treatments currently available. Their progression is fueled by persistent disruptions in hepatic metabolism, inflammation, and tissue remodeling. The autonomic nervous system, notably its regulatory role in hepatic function, is receiving increasing attention as a key mediator of pathogenesis of liver disease. Serotonin (5-hydroxytryptamine, 5-HT) has emerged as a pivotal regulator of all these processes. Acting via combined central and peripheral pathways and a range of receptor subtypes, 5-HT modulates hepatic glucose and lipid metabolism, immune responses, fibrogenesis, and liver regenerative capacity. In this review, we explore the multifaceted role of 5-HT signaling in metabolic control, obesity, liver physiology, and chronic liver diseases. Our focus extends beyond the direct effects of 5-HT on liver cell populations to its interaction with the autonomic nervous system, metabolic hormones, and the gut-liver-brain axis. Lastly, we discuss how 5-HT's dual origin and pleiotropic effects may offer therapeutic avenues for MASLD, ALD, and HCC.

## Introduction

**Figure 1:**
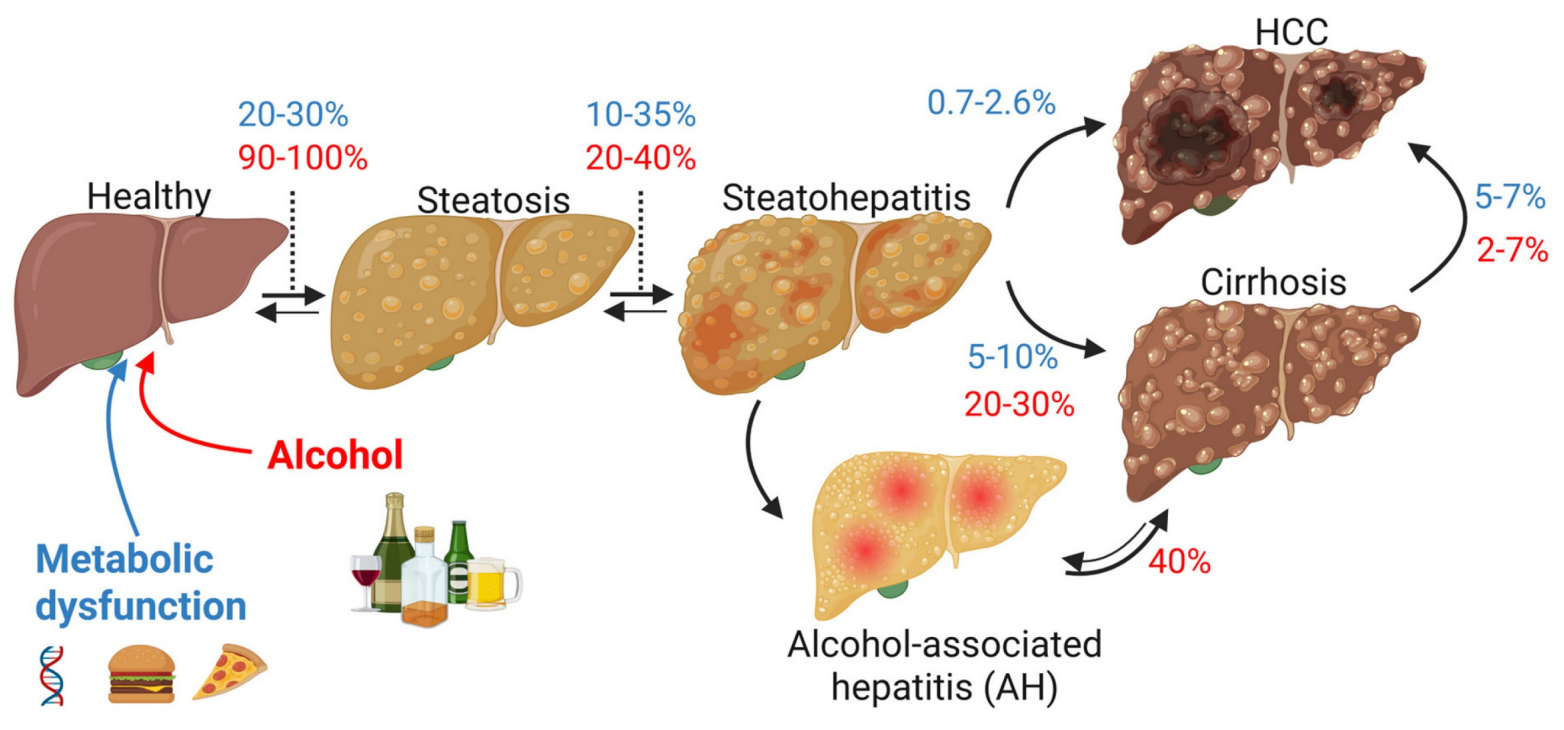
** Progression of steatotic liver diseases.** Steatotic liver diseases are associated with metabolic dysfunction (MASLD, blue) (Worldwide prevalence of 32.4%), chronic alcohol use (ALD, red) or both (MetALD), characterized by the progression from healthy liver to steatosis, steatohepatitis, and/or alcoholic hepatitis, that can both evolve in mild to severe fibrosis (cirrhosis) and ultimately lead to hepatocarcinoma (HCC). Blue percentages indicate estimated incidence of progression for MASLD, red for ALD. Double arrows show potential reversibility in the progression of the pathology. *Created with BioRender.com*.

Obesity and excessive alcohol intake are two major contributors to chronic liver diseases, notably metabolic dysfunction-associated steatotic liver disease (MASLD, previously NAFLD) and alcohol-associated liver disease (ALD) 1. MASLD, which closely mirrors the rising prevalence of obesity, type 2 diabetes, and metabolic syndrome, encompasses a spectrum of hepatic conditions from steatosis to steatohepatitis (MASH), fibrosis, and cirrhosis, and may ultimately progress to hepatocellular carcinoma (HCC) 1. Similarly, ALD evolves through stages of fatty liver, steatohepatitis (ASH), fibrosis, and cirrhosis, with a substantial risk of HCC in advanced disease stages, often compounded by episodes of acute-on-chronic liver injury 1. Indeed, patients with underlying ASH or cirrhosis and recent excessive alcohol consumption can develop alcohol-associated hepatitis (AH). Although MASLD and ALD originate from distinct etiologies-metabolic versus toxic-their downstream pathological mechanisms, including lipid accumulation, mitochondrial dysfunction, inflammation, fibrogenesis, and carcinogenesis, share striking similarities (**Figure 1**).

In the context of the search for common regulatory mechanisms, 5-HT has gained considerable attention for its emerging role in liver physiology and disease 2,3. While classically studied as a central neurotransmitter involved in mood, appetite, and behavior, over 90% of total body 5-HT is actually produced in the periphery, primarily by enterochromaffin cells of the gastrointestinal tract 4. Once synthesized, gut 5-HT is released into the bloodstream where it is stored in platelets and acts on several organs 5, including the liver 6. This dual origin, central and peripheral, adds a layer of complexity to 5-HT signaling, particularly in the context of MASLD and ALD.

Further complexity arises from the broad diversity of 5-HT receptors, with at least 14 subtypes grouped into seven families (5-HT_1_ to 5-HT_7_) 7. These receptors are variably expressed across liver cell types, including hepatocytes, hepatic stellate cells (HSC), cholangiocytes, endothelial cells, and immune cells, and mediate a wide range of effects from lipid metabolism and cell proliferation to inflammation, fibrogenesis, and even tumor progression 2. Growing evidence also implicates serotonergic signaling in the tumor microenvironment, supporting angiogenesis and cell proliferation in HCC 8. In parallel, central 5-HT, by acting through neuroendocrine and autonomic pathways, indirectly influences liver function, particularly in the context of energy homeostasis and metabolic control 9,10. This connection to the autonomic nervous system (ANS) is particularly important as the liver is densely innervated by both sympathetic and parasympathetic fibers 11. The ANS governs a wide array of hepatic functions, such as glucose production, bile secretion, and immune surveillance through efferent signals from the central nervous system 11. Central 5-HT modulates the activity of hypothalamic and brainstem nuclei that control autonomic output 12, thereby influencing liver physiology. Conversely, afferent vagal signaling from the liver to the brain contributes to central sensing of metabolic status 13, creating a bidirectional communication axis.

Altogether, 5-HT serves as a key integrator of gut, brain, and liver functions, acting both locally and systemically through an intricate network of serotonergic and autonomic pathways. Disentangling the respective contributions of peripheral versus central 5-HT, and their interaction with the ANS, are critical to understanding how serotonergic dysregulation contributes to the pathogenesis of MASLD and ALD. In this review, we aim to unravel the complex role of 5-HT in liver physiology and pathology, with a particular emphasis on its integration within the gut-liver-brain axis and its interconnection with other metabolic organs. We review the distinct sources of 5-HT, laying the necessary groundwork to differentiate the respective contributions of central versus peripheral 5-HT in the regulation of liver functions. This framework allows us to explore how disruptions in 5-HTergic signaling, from synthesis, degradation, receptor expression, to downstream pathways, can contribute to the onset and progression of chronic liver diseases, such as MASLD and ALD.

## I. Multiple sources of 5-HT

**Figure 2:**
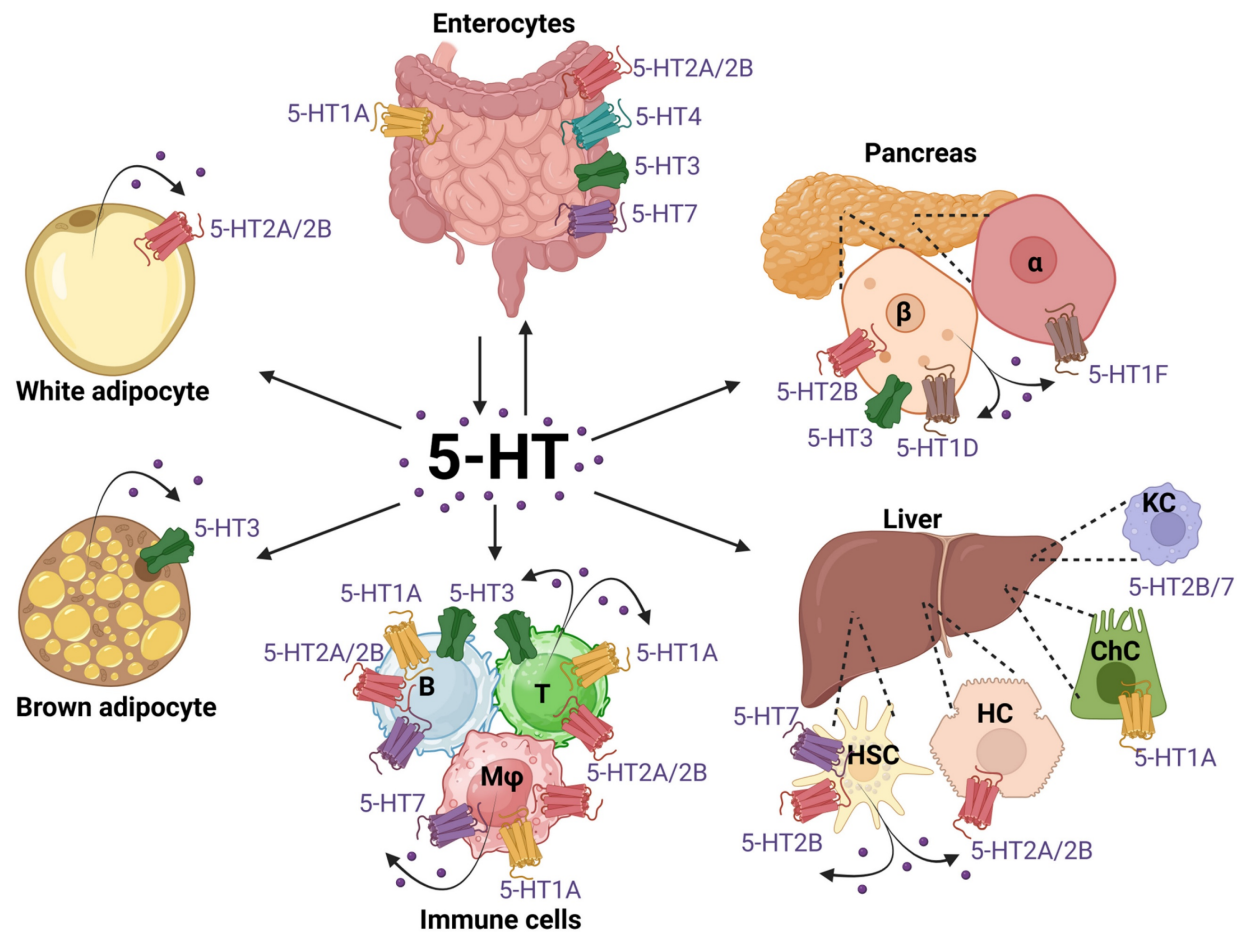
** Expression of peripheral 5-HT receptor subtypes across metabolic and immune systems.** The diagram illustrates the cell-specific expression of 5-HT receptors in key peripheral tissues involved in metabolic regulation: gut (enterocytes), liver (**HC**, hepatocytes; **HSC**, hepatic stellate cells; **ChC**, Cholangiocytes; **KC** Kupffer Cells), pancreas (**α** and **β** cells), white and brown adipose tissues, and in the immune system (**B,** B cells; **T,** T cells and **Mϕ**, macrophages. Multiple 5-HT receptor subtypes (e.g., 5-HT_1A/1D/1F_, 5-HT_2A/2B_, 5-HT_3_, 5-HT_4_, 5-HT_7_) are differentially expressed depending on the cell type, highlighting the widespread and pleiotropic roles of serotonin in peripheral physiology. *Created with BioRender.com*.

**Figure 3:**
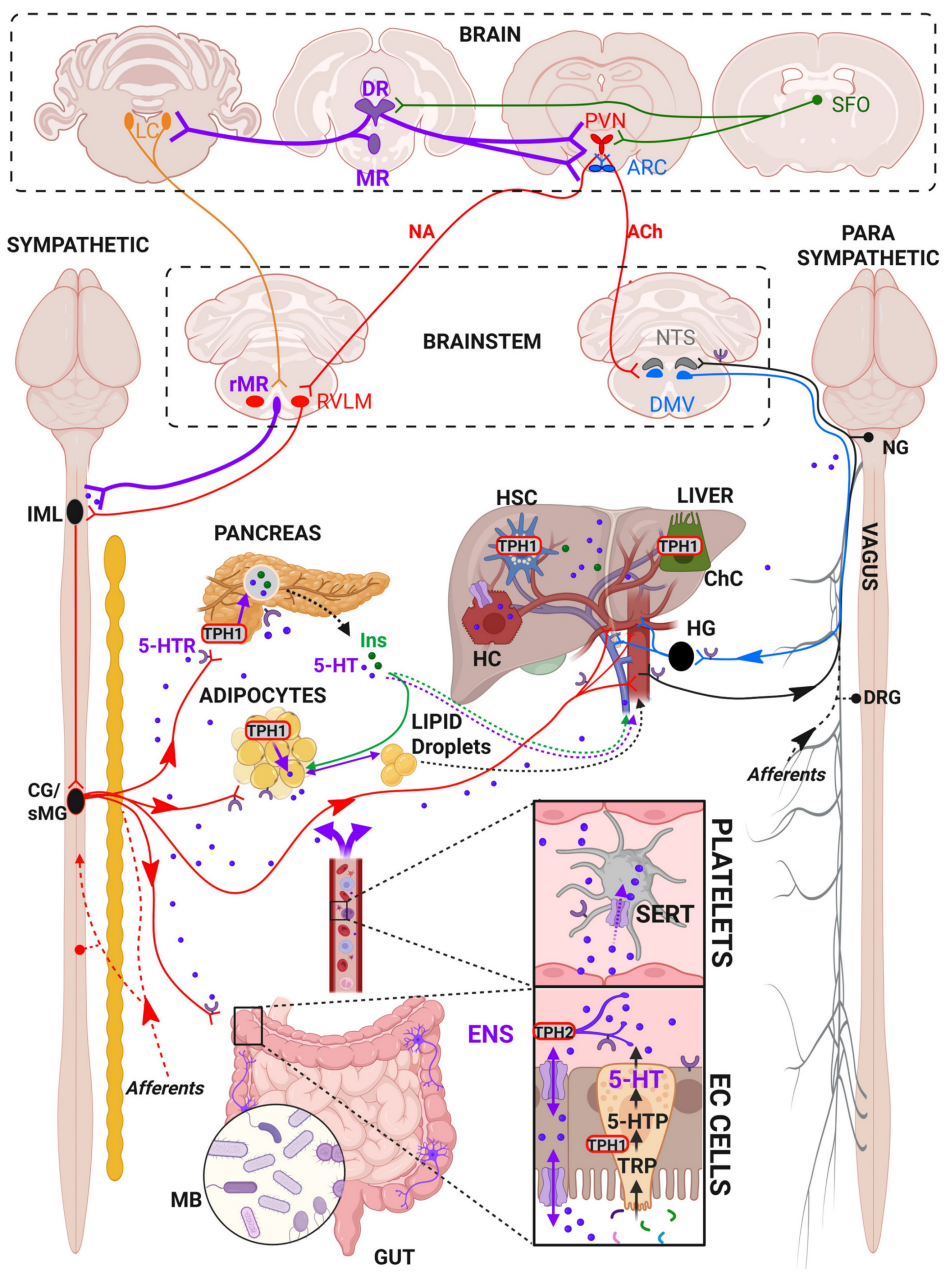
** Central and peripheral sources of 5-HT in the regulation of metabolic functions**. In the central nervous system, 5-HT is synthesized in the raphe nuclei (dorsal raphe, DR; median raphe, MR; rostral median raphe, rMR) by the tryptophan hydroxylase 2 (TPH2). 5-HTergic raphe (5-HT), noradrenergic locus coeruleus (LC), and subfornical organ (SFO) neurons are interconnected with the hypothalamic paraventricular nucleus (PVN) and the arcuate nucleus (ARC) to modulate autonomic sympathetic (rostral ventrolateral medulla, RVLM; Noradrenaline, NA) and parasympathetic preganglionic neurons (nucleus of the solitary tract, NTS; dorsal motor nucleus of the vagus, DMV; Acetylcholine, ACh) in the brainstem. RVLM and rMR neurons project through the spinal cord via the intermediolateral nucleus (IML) to the sympathetic coeliac (CG) and superior mesenteric ganglions (sMG), that innervate the different organs, including the liver, pancreas, adipose tissue, and gut. DMV neurons project to various parasympathetic ganglions, including hepatic ganglia (HG) via the descending vagal nerve. 5-HT can modulate the descending and ascending (afferents, sensory) sympathetic and parasympathetic branches via 5-HT receptors (5-HTR). In the gut, 5-HT is synthesized in EC) cells by the TPH1, which hydroxylates the tryptophan (TRP) coming from the lumen into 5-hydroxytryptophan (5-HTP), further converted to 5-HT. The gut is also innervated by the enteric nervous system (ENS) that expresses TPH2. Enterocytes express the serotonin transporter (SERT) so they can uptake and release 5-HT from and to the lumen or lamina propria. Once released, 5-HT can reach the blood where it is uptaken by the platelets that also express the SERT. Upon activation, platelets can release 5-HT to the organs. By influencing 5-HT synthesis and transport, the gut microbiota (MB) can modulate the levels of 5-HT produced by the gut. The pancreas expresses TPH1 and releases 5-HT together with insulin (Ins). Adipocytes also express TPH1 and synthetize 5-HT. Ins and 5-HT regulate lipolysis in the adipose tissues, which release lipid droplets that can accumulate in the liver. In the liver, only hepatic stellate cells (HSC) and cholangiocytes (ChC) produce 5-HT as they express the TPH1. Hepatocytes (HC) can store 5-HT as they express the SERT. *Created with BioRender.com*.

In mammals, the vast majority of 5-HT is produced in the gastrointestinal tract by enterochromaffin (EC) cells, in response to gut mechanical stimuli, nutrients (e.g., glucose, amino acids, short-chain fatty acids) or microbiota metabolites 4. It has been evaluated that 90% of 5-HT is synthesized in EC cells, by the rate-limiting enzyme tryptophan hydroxylase 1 (TPH1) 14, which converts tryptophan into 5-hydroxytryptophan, further converted to 5-Hydroxytryptamine by the aromatic amino acid decarboxylase (AADC). Gut microbiota influences TPH1 activity and 5-HT production in the gut through multiple mechanisms 15. While certain microbial species can upregulate the expression of TPH1 and enhance local 5-HT synthesis - particularly through metabolites such as short-chain fatty acids (SCFAs) including acetate, butyrate, and propionate - others can suppress TPH1 expression via the production of secondary bile acids or tryptamine derivatives, thereby reducing mucosal 5-HT synthesis 16-19. Other microbial species can synthesize tryptophan (providing more substrate for TPH1-mediated 5-HT synthesis), or express the AADC, hence contributing to 5-HT production 20. Through these actions, the microbiota plays a pivotal role in modulating 5-HT availability in the inflamed gut and/or in a context of obesity 21. In turn, by modulating the growth of certain bacteria, or reducing tryptophan availability due to its synthesis, 5-HT may shape gut microbiota composition to stimulate inflammatory and immune responses 22. Once secreted by EC cells, 5-HT is either acting locally in a para- or autocrine manner or it is released into the systemic circulation, as a hormone. In the bloodstream, most of the 5-HT is stored in blood platelets 23. Upon platelet activation, 5-HT is released into various organs, including the liver, pancreas, and adipose tissue, thereby regulating their homeostasis or metabolic activity 24 through activation of multiple 5-HT receptors (**Figure 2**). Cells expressing TPH1 and capable of synthesizing 5-HT are also found in the liver (i.e., stellate cells and cholangiocytes) 25,26, pancreas (β-cells) 27, and adipose tissue (adipocytes) 28, suggesting an additional local source of 5-HT in those organs (**Figure 3**).

Another important source of 5-HT is the nervous system, where it is synthesized by the tryptophan hydroxylase 2 (TPH2) in lower relative quantities and serves as a neurotransmitter. 5-HT is present in the enteric neurons (ENS) of the peripheral nervous system (PNS), where it regulates intestinal motility and anxiety 29,30, and in the raphe neurons of the brainstem within the central nervous systems (CNS), where it regulates a wide range of physiological, psychological, and cognitive functions such as appetite, sleep, mood, fear, reward, learning, and memory 31.

From these different sources, 5-HT can further influence most of the metabolic functions controlled by the autonomous nervous system. 5-HT regulates glucose and lipid homeostasis, appetite and satiety, energy expenditure, and thermogenesis by modulating the sympathetic and parasympathetic activity in the liver, the pancreas or the adipose tissues 9. For instance, 5-HT neurons from the rostral medullary raphe (rMR) indirectly project to the coeliac (CG) and superior mesenteric (sMG) ganglions that relay sympathetic innervation to the liver 32-34, pancreas 32,35, and adipose tissues 32,36. There is also evidence showing that 5-HT immunoreactive axons are detectable within sympathetic nerves and could directly innervate sympathetic ganglions 37-43 to modulate sympathetic outflow 44,45. In addition, the descending parasympathetic (efferent) transmission of the vagal nerve, which originates from the dorsal motor nucleus of the vagus (DMV) and innervates various organs, is also facilitated by 5-HT, an effect probably mediated by 5-HT_3_ receptors located on vagal nerve terminals 46,47. The resulting stimulation of both descending sympathetic 48,49 and parasympathetic 50,51 pathways can modulate the activity of ENS 5-HT neurons, which would facilitate 5-HT release from EC cells 52 (**Figure 3**). In turn, circulating 5-HT may stimulate the sympathetic (spinal) and parasympathetic (vagal) sensory afferents, thereby forming a feedback loop within the brain-gut axis 53. Furthermore, 5-HT neurons from the dorsal raphe nuclei (DRN) directly project to the nucleus of the tractus solitary (NTS) 54,55 in the medulla to further modulate the ascending parasympathetic (afferent) transmission of the vagal nerve coming from the liver 32,34, pancreas 32,35 and adipose tissues 56-59 (**Figure 3**). Interestingly, 5-HT producing neurons have been found within the sensory nodose ganglion (NG) of the vagus nerve 60,61 and the NTS 62, with a population of 5-HT immunoreactive neurons projecting from the nodose ganglion to the NTS 63,64, although it is unclear whether they release 5-HT in a physiologically-relevant manner 65. This suggests that 5-HT innervation is found along both the sympathetic and parasympathetic systems to modulate the autonomic afferent and efferent signaling from and to various organs. Finally, some evidences have demonstrated sparse 5-HT innervation in liver, around the portal vein, portal artery, bile duct, and within hepatic lobules 66-68, suggesting that 5-HT regulates hepatic blood flow 69,70 but could also modulate hepatocellular functions.

Together, this highlights the intricated connections between central and peripheral 5-HT and supports the idea that a precise control of 5-HT levels is essential for liver physiology and related organs' metabolic functions. For example, traumatic brain injury and neurodegenerative diseases (Parkinson, Alzheimer) are associated with reduced peripheral 5-HT signaling 71-73, decreased lipogenesis, lipid accumulation in liver and adipose tissues, and alterations in the gut microbiota composition 71,74.

## II. Central 5-HT Signaling and Liver Physiology

### a. Regulation of liver physiology by central 5-HT

The main function of the liver is to regulate metabolism, detoxification, and energy homeostasis. Hepatic cytochrome P450 (CYP) monooxygenases play a key role in the metabolism of endogenous substances such as steroids, fatty acids, and neurotransmitters, as well as xenobiotics like drugs and toxins (for review see 75). Beyond its metabolic and detoxifying functions, the liver is a major immune organ responding to exogenous antigens, metabolites, and molecular patterns. In obesity, sentinel cells such as liver-resident macrophages (Kupffer cells) and monocyte-derived macrophages sense the persistent accumulation of metabolites, antigens, and pattern molecules. This recognition shifts the liver from an immune-tolerant to an activated immune state, with reduced anti-inflammatory cytokines (TGF-β, IL-10) and increased pro-inflammatory cytokines (IL-1, IL-6, TNF-α). The resulting interplay between immune cells and hepatocytes sustains low-grade inflammation in MASH, while liver sinusoidal endothelial cells (LSECs), macrophages, innate lymphoid cells (ILCs), and neutrophils further contribute to disease progression and fibrosis (for review see 76). A fine regulation of these hepatic functions must therefore be in place, in part through the nervous system, to coordinate appropriate responses.

The regulation of hepatic CYP enzymes is primarily controlled by endocrine signals, particularly growth hormone (GH), glucocorticoids (GR), thyroid hormones, and sexual hormones that act via nuclear receptors to modulate CYP gene expression 77. The brain 5-HTergic system seems to play a significant role in the neuroendocrine regulation of hepatic CYP activity. Indeed, 5-HT projections from the DRN and median raphe nuclei (MRN) innervate the paraventricular (PVN) and arcuate (ARC) nuclei of the hypothalamus, key centers for hormone regulation 77,78. Selective depletion of 5-HT innervation in the ARC or PVN by neurotoxic lesioning, which reduces 5-HT levels in these areas, produces opposite effects, with ARC-5-HT depletion decreasing, and PVN-5-HT depletion increasing CYP2C11 expression and function 79. This data suggests that 5-HT exerts an opposite, region-specific effect on liver CYP enzymes, depending on the hypothalamic nucleus involved. Interestingly, depletion of central 5-HT in DRN and MRN by genetic ablation, neurotoxic lesioning or following a tryptophan-deficient diet, increases the liver expression and activity of CYP1A1/A2, CYP2C11, and CYP3A1 80-82, suggesting that 5-HT acts predominantly as a negative regulator of these enzymes. In line with this, stimulation of brain 5-HT by chronic intracerebroventricular administration of its precursor 5-HTP decreases the expression and activity of liver CYP1A2, CYP2A2, CYP1B, CYP2C11, and CYP3A1/2 78*.*

Bidirectional 5-HTergic control of CYP function is likely mediated by 5-HT_1A_ receptors in the PVN and 5-HT_2C_ receptors in the ARC. Stimulation of 5-HT_2C_ receptors (excitatory) in the ARC increases the activity and expression of CYP2C11, CYP3A1/23, and CYP3A2*,* and is associated with an increase in the pituitary growth hormone-releasing hormone (GHRH) and serum GH levels 83. Conversely, stimulation of the 5-HT_1A_ receptors (inhibitory) in the PVN suppresses GH secretion, leading to a reduction in the expression and activity of CYP2C11 and CYP3A1 in the liver 77. This effect is attributed to 5-HT-mediated stimulation of somatostatin release in the hypothalamus, which inhibits GH secretion and downregulates GH-dependent CYP enzymes 78. Together, these data suggest that 5-HT exerts an opposite and region-specific regulatory effect on liver CYP enzymes, depending on the hypothalamic nucleus involved.

**Figure 4:**
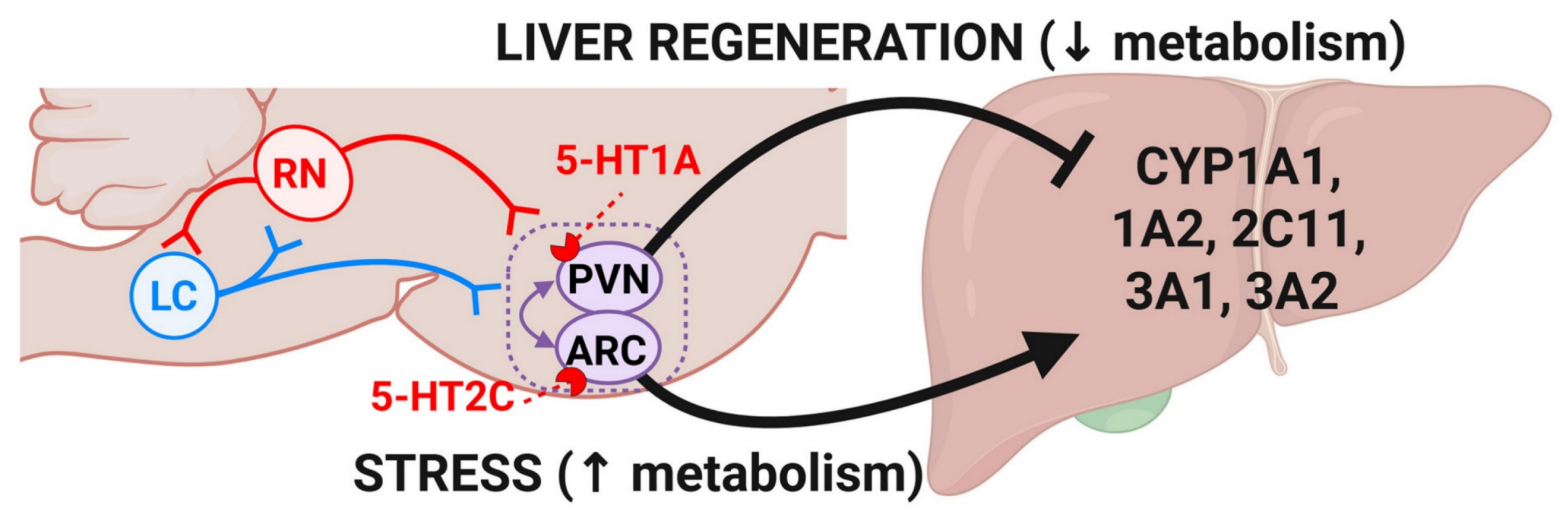
** Central 5-HT modulation of hepatic cytochrome activity orchestrates the balance between liver regeneration and metabolic processes.** Together with their interplay with the brainstem adrenergic locus coeruleus (LC), the 5-HTergic raphe nuclei (RN) regulate both the hypothalamic paraventricular nucleus (PVN) via 5-HT_1A_ receptors to inhibit hepatic metabolism during liver regeneration, and the arcuate nucleus (ARC) via the 5-HT_2C_ to stimulate hepatic metabolism in response to stress. 5-HT_1A_ signaling in the PVN reduces, while 5-HT_2C_ signaling in the ARC stimulates CYP1A1, CYP1A2, CYP2C11, CYP3A1, and CYP3A2 expression or activity.* Created with BioRender.com*.

Noradrenaline (NA) plays a complementary role in hepatic CYP regulation, with the locus coeruleus (LC, the major noradrenergic center) projecting to the PVN and ARC nuclei influencing GH and other pituitary hormones 84. Lesioning of the LC, which reduces NAergic input, alters GH secretion and increases hepatic CYP2C11 expression 84. These findings indicate that, like serotonin, norepinephrine modulates CYP activity through neuroendocrine mechanisms. Using a combination of anatomical tracing and “chemogenetics”, a recent report has identified a brain-to-liver neural circuit that inhibits liver regeneration following chronic stress. This pathway involves NA neurons in the locus coeruleus (LC) projecting to 5-HT neurons in the medullary raphe nucleus (mRN), which modulates sympathetic innervation to the liver. This circuit becomes hyperactive under chronic stress, leading to sustained NE release in the liver, which suppresses pro-inflammatory macrophage activation and inhibits hepatic regenerative processes 85. These findings suggest that brain 5-HT signaling could mediate the inhibitory action of stress on hepatic regeneration. Interestingly, an upregulation of most CYP isozymes and hepatic metabolism is also observed following chronic exposure to psychological stress (for review see 86,87), whereas CYP downregulation is observed during liver regeneration 88. Taken together, this data suggests that central 5-HT (together with the interplay with central NE) could regulate the balance between the metabolic and the regenerative activity of the liver 89,90, by adapting liver function to physiological demands, stress, and injury 91,92. Since transient hepatic lipid accumulation (steatosis) is shown to be essential for regeneration after hepatectomy, it supports the idea that central 5-HT signaling could modulate the regenerative process by controlling hepatic lipid metabolism 93 (**Figure 4**).

### b. Role of central 5-HT in the regulation of metabolic hormones

**Figure 5:**
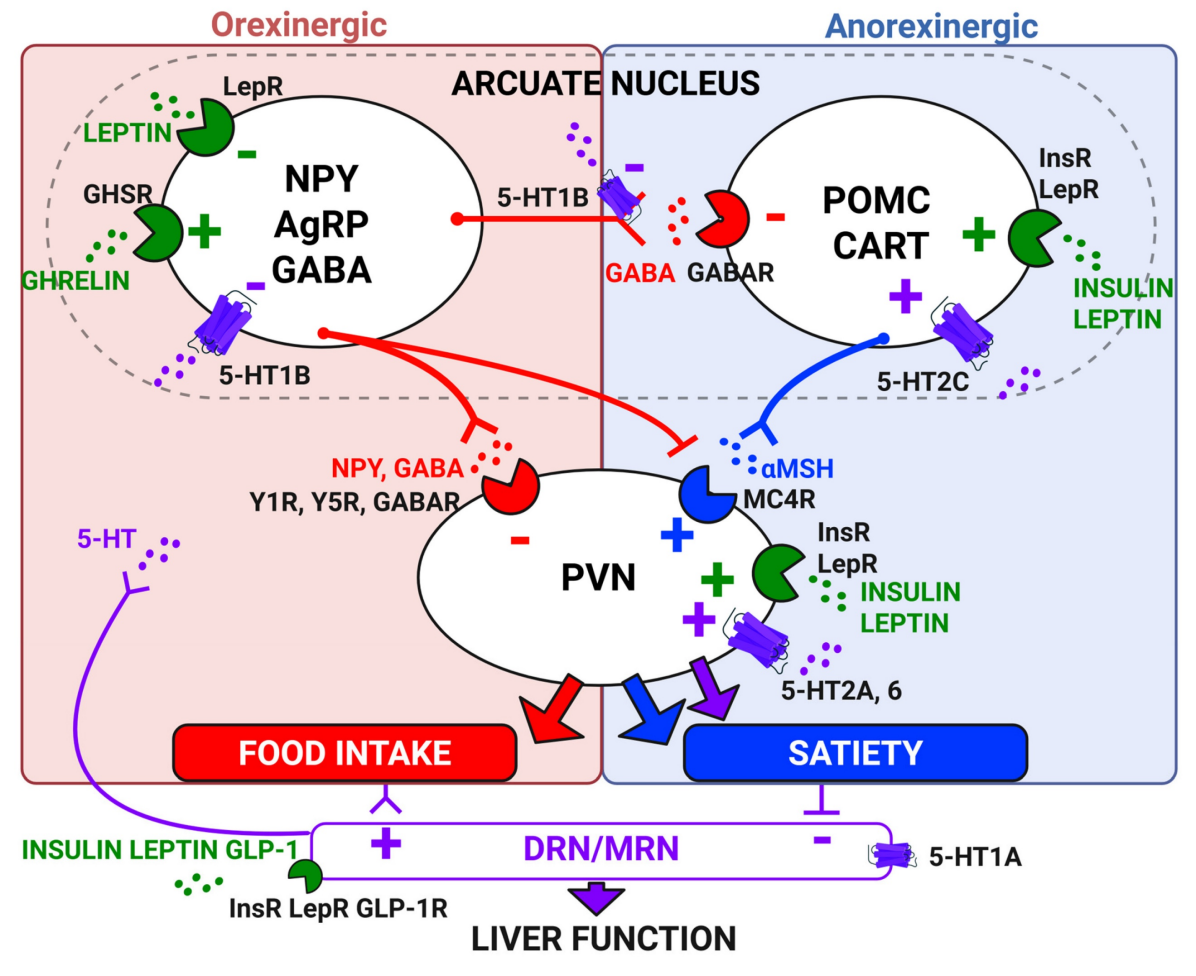
**Modulatory role of central 5-HT on hypothalamic metabolic hormones, food intake and liver function.** Within the hypothalamus, the arcuate nucleus (ARC) and the paraventricular nucleus (PVN) are interconnected to regulate food intake via the orexinergic pathway (light red) and satiety via the anorexinergic pathway (light blue). Pro-opiomelanocortin (POMC) and cocaine- and amphetamine-regulated transcript (CART) neurons release α-melanocyte-stimulating hormone (α-MSH), which activates melanocortin 4 receptors (MC4R) in the PVN to promote satiety and suppress food intake. In contrast, ARC orexinergic neurons release neuropeptide Y (NPY), agouti-related peptide (AgRP), and gamma-aminobutyric acid (GABA), which inhibit the anorexigenic effects of POMC neurons on the PVN, thereby suppressing satiety signals and promoting feeding. These effects are mediated through inhibitory GABA receptors (GABA_R_) and NPY receptors (Y1 and Y5) on POMC and PVN neurons. Food intake activates the DRN and MRN, leading to the release of 5-HT in the ARC and PVN. This serotonergic input inhibits feeding via activation of NPY-5-HT_1B_ receptors and promotes satiety via stimulation of POMC-5-HT_2C_ and PVN-5-HT_2A,6_ receptors. Conversely, in the absence of food, DRN/MRN activity is suppressed through 5-HT_1A_ receptor signaling, leading to reduced 5-HT release in the ARC and PVN, thereby lifting the inhibition on feeding. Peripheral metabolic hormones also modulate these central circuits: insulin, leptin, and GLP-1 exert anorexigenic effects through their respective receptors (InsR, LepR, and GLP-1R) located on DRN/MRN, ARC, or PVN neurons. In contrast, ghrelin exerts orexigenic effects by stimulating its receptor (GHSR) on NPY neurons. This complex interplay between central serotonergic pathways, hypothalamic feeding circuits, and peripheral metabolic hormones enables fine-tuned regulation of feeding and satiety, while also contributing to the homeostatic control of liver function in response to nutritional states. *Created with BioRender.com*.

Beyond its classical role in the brain, central 5-HT plays a pivotal role in systemic metabolic regulation, in part through its intricate interactions with a range of key metabolic hormones-including insulin, leptin, ghrelin, glucagon, glucagon-like peptide 1 (GLP-1), glucocorticoids, and thyroid hormones-which collectively influence liver function and energy homeostasis (**Figure 5**).

**Insulin** is the hormone secreted by pancreatic β-cells in response to high blood glucose levels. Once secreted by the pancreas in response to food intake, insulin stimulates the storage of glucose and lipid in the liver 94,95 and suppresses appetite by stimulating pro-opiomelanocortin neurons on the ARC. Insulin can further increase the firing activity of DRN 5-HT neurons by direct activation of insulin receptors located on 5-HT neurons 96. In turn, chronic DRN neuron activation or chronic intranasal administration of 5-HT improves hepatic lipid metabolism, reduces liver steatosis, and ameliorates systemic (muscle) tolerance to glucose, and hepatic and systemic (muscle) sensitivity to insulin 93,97. This therefore suggests a potential role of central 5-HT deficiency in insulin resistance and metabolic dysfunctions 98.

**Leptin** is a satiety hormone primarily produced by adipose tissues in response to increased lipid storage, which signals the hypothalamus to reduce appetite and to increase energy expenditure. Leptin can act directly or indirectly (by stimulating hypothalamic PVN 99) on the liver to inhibit gluconeogenesis, stimulate glycogenolysis, increase insulin sensitivity, inhibit lipogenesis, reduce triglyceride secretion, and stimulate fatty acid oxidation 100-103. By preventing lipid accumulation in the liver, leptin is considered an anti-steatotic hormone 100,101,104 although exerting a pro-fibrotic action 105. Hence, leptin resistance may occur rapidly and be exacerbated in MASLD 100,106. DRN 5-HT neurons projecting to the ARC express the leptin receptor and the resulting activation of DRN-ARC by leptin inhibits feeding behaviors 107. Conversely, DR 5-HT depletion prevents the effect of leptin on the reduction of food intake 108.

**Ghrelin** is an appetite stimulating hormone released by the empty stomach that acts through its receptor, growth hormone secretagogue receptor (GHSR), on liver and PVN neurons to promote food intake, stimulate hepatic glucose production, and inhibit insulin signals 109. Whether ghrelin stimulates 110,111 or inhibits 112,113 5-HT neurons activity is not clearly established yet. However, it is likely that ghrelin and 5-HT have opposite effects on food intake. Hence, intra-PVN injections of 5-HT antagonize the orexinergic effect of ghrelin 114, probably via activation of 5-HT_2C_ receptors on pro-opiomelanocortin (POMC) neurons 115. Conversely, inhibition of MRN activity by the stimulation of 5-HT_1A_ receptors potentiates the orexinergic effect of ghrelin injection into the PVN 116. This suggests that 5-HT and ghrelin could exert an opposite action on liver metabolic functions.

**Glucocorticoids** (GC) are secreted by the adrenal cortex and delivered to the liver via the bloodstream. Through activation of the glucocorticoid receptor (GR), GC influence many important liver functions such as gluconeogenesis, glucose uptake and utilization 117, *de novo* lipogenesis, lipid export, fatty-acid oxidation 118, and insulin sensitivity 119. Thus, it has been suggested that chronic alterations in GC/GR signaling could lead to MASLD 120. The role of 5-HT as an activator of the hypothalamus-pituitary-adrenal axis (HPA) and the subsequent release of adrenal GC is well established 121,122. As part of a negative feedback loop, GC reduces 5-HT release in the PVN 123. This suggests that dysregulation of the 5-HT/GC loop could be associated with alterations in liver functions.

**Glucagon** is a hormone released by pancreatic α-cells in response to low blood glucose levels to mobilize energy. By acting directly on the liver, glucagon promotes hepatic glucose production through stimulation of glycogenolysis and gluconeogenesis, while inhibiting glycolysis 124. It also reduces hepatic lipid accumulation and secretion, adiposity, and body weight 125-128. Glucagon may therefore reduce liver steatosis, and glucagon receptor agonists are currently being developed as a therapeutic strategy for MASH/MASLD 129. The release of glucagon by the pancreas seems to be controlled by glucose-sensing neurons in the hypothalamus and the brainstem through the autonomic nervous system (ANS) 130,131. In turn, the glucagon released by the pancreas reaches the brain to regulate hypothalamus and brainstem functions to lower hepatic glucose production and decreases food intake 132-134. Although the interconnection between glucagon and central 5-HT has not been established yet, it could occur via their respective signaling in the hypothalamus and/or the brainstem. Locally in the liver, both synergic and opposite cross-talks exist between glucagon and 5-HT receptors: 5-HT_2B_ and 5-HT_7_ receptors potentiate glucagon's hyperglycemic action by promoting gluconeogenesis, whereas 5-HT_1A_ and 5-HT_2A_ receptors antagonize glucagon's hyperglycemic action by stimulating glycogenesis.

**Glucagon-like peptide 1** (GLP-1) is an incretin hormone produced from the same precursor as glucagon (proglucagon) by the intestine and the nucleus of the solitary tract (NTS) in the brainstem, in response to glucose and lipid. Intestinal GLP1 can either be secreted to reach hypothalamus via the blood circulation 135, act locally to stimulate gut 5-HT release 136 or activate the enteric nervous system that projects to NTS preproglucagon neurons which releases GLP1 in the hypothalamus. This combined effect modulates the autonomic nervous system to stimulate insulin secretion, which lowers hepatic glucose production 137, enhances hepatic glycogen storage 138, negatively regulates food intake, and reduces body weight 139. Therefore, GLP1 receptor agonists have been proposed in the treatment of MASLD 140-144. NTS GLP1 neuron activity is modulated by central 5-HT via 5-HT_1A_ and 5-HT_2C_ receptors 145. Interestingly, the hypophagic effect of 5-HT_2C_ receptor agonists is mediated by NTS GLP1 neurons 146. In turn, GLP1 acts in the DRN to modulate central 5-HT release and reduce appetite and body weight 147,148.

**Insulin-like growth factor 1** (IGF-1), is a hormone produced by the liver, with a molecular structure similar to insulin, that plays important roles in the development and growth during childhood, and has, to a lesser extent, metabolic effects such as stimulation of glucose uptake and lipid metabolism, and reduction of blood glucose in adults. In the liver, IGF-1 likely reduces steatosis, fibrosis, and the overall severity of MASLD 149. 5-HT and IGF-1 bidirectionally interact within the brain, particularly in regions such as the hypothalamus, including the ARC and PVN. On one hand, 5-HT positively regulates IGF-1 by stimulating hypothalamic GHRH, which promotes growth hormone (GH) secretion from the anterior pituitary, thereby enhancing hepatic IGF-1 synthesis 150. On the other hand, IGF-1 influences 5-HT neurotransmission, by stimulating serotonergic input from the DRN to the hypothalamus, particularly in the PVN and ARC, potentially influencing appetite, energy balance, and stress responses 151.

**T3 and T4 thyroid hormones** (TH) release from the thyroid gland is controlled by the thyroid stimulating hormone (TSH) secreted by the pituitary, itself under the control of the thyrotropin-releasing hormone (TRH) produced by the PVN. T3 and T4 exert negative feedback on both TSH and TRH secretion. By acting directly on the liver and indirectly via the PVN, TH regulates lipid and glucose homeostasis, through cholesterol modulation and fatty acid synthesis, increased lipolysis, lipid droplets formation, free fatty acid uptake, glucose production, and reduced insulin sensitivity 152. In turn, T3 suppresses both hypothalamic 5-HT activity and TSH secretion, indicating direct negative feedback at the hypothalamic level and suggesting that the 5-HT/thyroid interplay regulates liver physiology.

### c. Role of the autonomic nervous system in the regulation of liver functions and diseases: potential modulation by 5-HT

The autonomic nervous system, comprising the sympathetic and parasympathetic branches, serves as a crucial interface between the brain and peripheral organs, orchestrating the regulation of systemic metabolism through dynamic neural control of energy balance, inflammation, and organ-specific functions.

The sympathetic nervous system (SNS) plays a pivotal role in hepatic lipid and glucose metabolism, especially under metabolic stress conditions. High-fat diet challenge, liver steatosis, aging, and metabolic syndrome are associated with altered liver sympathetic tone, leading to changes in intrahepatic noradrenaline (NA) signaling 153-165. This adrenergic signaling upregulates fatty acid transporters (CD36) and lipogenic enzymes (DGAT1 and DGAT2) 166, which accelerates hepatic triglyceride accumulation and steatosis 153. Notably, liver sympathetic denervation is shown to reverse obesity-induced hepatic steatosis, supporting the role of the SNS in lipid accumulation 166,167. Beyond lipid metabolism, NA also exerts significant control over glucose handling in the liver. It stimulates gluconeogenic pathways through β2-adrenergic receptor-mediated activation of glucose-6-phosphatase and phosphoenolpyruvate carboxykinase, while suppressing glycogenesis 168,169. These effects promote hepatic glucose output, contributing to hyperglycemia and the development of liver insulin resistance, which in turn exacerbates *de novo* lipogenesis and steatosis 170,171. In addition, the SNS negatively regulates liver regeneration by preventing hepatocyte priming and the entry into the cell cycle after partial hepatectomy 172.

In parallel, sympathetic input enhances the pro-inflammatory state by promoting Kupffer cell cytokine production, particularly tumor necrosis factor-alpha (TNF-α) and interleukin-6 (IL-6), thereby pushing the progression from steatosis to steatohepatitis 173,174. Furthermore, the SNS directly contributes to liver fibrogenesis. NA and neuropeptide Y (NPY) activate HSC through α- and β-adrenergic receptors, triggering PI3K/MAPK pathways that drive HSC proliferation, α-smooth muscle actin (α-SMA) expression, and extracellular matrix synthesis 175-177. Clinically, elevated plasma NA and increased sympathetic tone are observed in cirrhotic patients, correlating with portal hypertension and fibrosis severity 178. However, sympathetic integrity also appears essential for adequate liver repair and regeneration, with loss of hepatic sympathetic fibers being associated with metabolic dysfunction and impaired regenerative capacity 176,177,179,180.

Conversely, the parasympathetic nervous system (PNS), via the vagus nerve, plays a largely protective role in liver inflammation and metabolism 181,182. Acetylcholine (ACh) released from vagal efferents binds to α7-nicotinic ACh receptors (α7nAChRs) on Kupffer cells and hepatocytes, suppressing NF-κB activation and downstream transcription of pro-inflammatory cytokines such as TNF-α, IL-1β, and IL-6 183-185. This cholinergic anti-inflammatory effect has been shown to reduce steatohepatitis in multiple murine models 182. Pharmacological enhancement of vagal tone using galantamine, an inhibitor of the ACh degradation enzyme (acetylcholine esterase, AChE), reduces hepatic inflammation, steatosis, and systemic insulin resistance 186,187. Conversely, selective hepatic vagotomy results in exacerbation of steatohepatitis, with increased inflammatory cytokine expression, and a concomitant decrease in peroxisome proliferator-activated receptor alpha (PPARα) activity leading to the aggravation of liver steatosis 185. As a result, the PNS enhances liver insulin sensitivity and suppresses hepatic glucose output, serving as a metabolic counter-regulator to the SNS 170,188.

There is also some evidence supporting a fibrogenic role of parasympathetic innervation. For instance, parasympathetic modulation *in vitro* appears pro-fibrogenic with ACh promoting HSC proliferation and collagen I transcription via muscarinic M2 receptors 189,190. Hence, hepatic vagotomy reduces HSC proliferation 191 and fibrogenesis 192 in diet- and toxin-induced mouse models of liver fibrosis. Nevertheless, parasympathetic signals are implicated in liver regeneration, particularly through vagal efferents that modulate hepatocyte proliferation and liver progenitor activation 193,194.

Collectively, these findings highlight a vicious cycle wherein sympathetic overactivation promotes hepatic steatosis, insulin resistance, inflammation, and fibrosis, while parasympathetic activity mitigates these processes and supports regenerative outcomes. Disruption of this autonomic balance, promoting sympathetic tone and vagal stillness, may increase the risk of the onset and progression of chronic liver diseases 195,196. Interestingly, serotonin (5-HT) appears to mirror and potentially amplify the pathological actions of the sympathetic nervous system on liver metabolism and inflammation. Similar to noradrenaline, 5-HT exacerbates insulin resistance by stimulating hepatic glucose output and impairing insulin signaling 197, enhances liver steatosis via 5-HT_2A_ receptors on hepatocytes 198,199, and fibrogenesis (upregulation of α-SMA, TGF-β1, and extracellular matrix components) through activation of 5-HT_2A_ and 5-HT_2B_ receptors on hepatic stellate cells 200. In addition, 5-HT has emerged as a key modulator of liver regeneration, particularly via platelet-derived 5-HT which primes hepatocytes for cell cycle entry and regeneration following partial hepatectomy 201. 5-HT may also directly modulate autonomic nerve terminal activity within the liver since a large body of evidence demonstrates that 5-HT influences autonomic efferent activity by acting on various prejunctional 5-HT receptors, to either inhibit or facilitate autonomic neurotransmitter release, thereby affecting processes like heart rate, blood pressure, and gastrointestinal motility (for review see 202). Although such mechanisms remain unexplored in the liver, the dense autonomic innervation and the presence of multiple 5-HT receptor subtypes in liver raise the hypothesis that 5-HT could shape its function not only via hepatocytes or immune targets but also by modulating the activity of sympathetic and parasympathetic fibers. This putative serotonergic-autonomic interface may constitute a critical amplification loop.

Recent findings also highlight the pivotal role of brain-derived neurotrophic factor (BDNF) and its receptor TrkB in the maintenance of hepatic autonomic innervation, overall liver homeostasis, and insulin sensitivity 203-205. In humans and rodents, BDNF deficiency is associated with increased appetite and weight gain, higher glucose levels, and elevated risk of liver steatosis and steatohepatitis 206-209. In models of metabolic stress, such as HFD-induced MASH, liver tissue undergoes selective sympathetic neuropathy, driven by TNF-α-induced axonal degeneration 171. This neuropathic loss impairs noradrenergic tone and disrupts sympathetic regulation of hepatocyte function, contributing to metabolic imbalance and liver inflammation 171. On the other hand, BDNF levels are elevated in fibrotic liver tissues and appear to participate in both neuronal and fibrogenic signaling 210. Notably, by acting on its TrkB receptor, BDNF can activate hepatic stellate cells and the upregulation of pro-fibrogenic markers 210. In the meantime, it also plays a protective neurotrophic role via TGF-β/SMAD signaling 211, which may be essential for preserving liver innervation 212. Loss of neurotrophic support may therefore not only facilitate inflammation and fibrosis but also hinder the neuronal plasticity and reinnervation necessary for effective liver regeneration.

5-HTergic signaling and BDNF may also cooperate in regulating hepatic autonomic nerve plasticity and/or degeneration, as observed in the brain 213,214. Therefore, the progressive loss of autonomic nerve fibers observed in chronic liver disease may not only reflect neurodegeneration but also a failure of trophic and regenerative support, with 5-HT-BDNF co-signaling representing a key modulator in this process.

## III. Role of Peripheral Serotonin in Metabolic and Liver Diseases

### a. Obesity

Obesity is the most prevalent metabolic disease worldwide, affecting over 1 billion children and adults, approximately 13% of the global population and its prevalence continues to rise 215. It is a major driver of a wide range of metabolic disorders, including type 2 diabetes, cardiovascular disease, and MASLD. Peripheral 5-HT is an important pathogenic contributor to obesity and associated dysglycemia 216 and dyslipidemia 217. Human obesity is characterized by increased production and release of gut-derived 5-HT, which is strongly linked to abnormal glycemic control, altered lipid levels, and higher body mass 218**.** Similarly, increased 5-HT levels and TPH1 activity are consistently observed in animal models of obesity 28,219-224.

In mice fed a high-fat diet (HFD), the pharmacological blockade of 5-HT synthesis with TPH inhibitors, either parachlorophenylalanine (PCPA) or LP-533401, decreases body weight gain, improves glucose tolerance, and lowers adiposity 219,225. These effects are likely mediated by peripheral TPH1 because opposite effects (weight and body fat gain) are observed when inhibiting TPH2 by intracerebroventricular injections of PCPA 226. The link between 5-HT synthesis and obesity has been confirmed genetically. Mice depleted for TPH1 (*Tph1*^-/-^) are protected from HFD-induced obesity and related metabolic dysfunctions, showing less weight gain, lower adiposity, reduced insulin resistance, and liver steatosis compared to control mice 227. This work suggests that 5-HT-mediated obesity and metabolic dysfunction could be linked to local 5-HT synthesis by adipose tissues (AT). In mice fed an obesogenic diet, TPH1 expression and tissue 5-HT levels are augmented in white (W) and brown (B) AT 219,227. Selective ablation of TPH1 in adipose tissues reduces weight gain and improves glucose tolerance and insulin sensitivity after HFD 28,219,223. Reduced adiposity in epididymal and inguinal WAT and increased energy expenditure by BAT are also observed, similarly to PCPA-treated mice 219. This suggests that adipocyte-derived 5-HT promotes energy storage in WAT while inhibiting energy expenditure in BAT.

The effect of 5-HT on adipose tissues is likely mediated by various 5-HT receptors, including 5-HT_3_, 5-HT_2A_, and 5-HT_2B_ receptors. Mice genetically depleted for 5-HT_3_ receptors show reduced body gain, improved glucose tolerance, and increased BAT activity upon HFD challenge, suggesting a potential role of 5-HT_3_ receptors in thermogenesis 219. Increased mRNA expression of 5-HT_2A_ and 5-HT_2B_ receptors is observed in the visceral adipose tissue (VAT) of human patients and WAT of mice (leptin deficient *ob/ob*, HFD) suffering of obesity 228, suggesting that alterations in the expression of these receptors contribute to AT expansion. Indeed, mice with selective knockout of 5-HT_2A_ receptors in adipose tissues have reduced *de novo* lipogenesis in WAT, improved glucose tolerance, and are resistant to HFD-induced obesity 228. On the other hand, 5-HT_2B_ receptors seem involved in fatty acids lipolysis and free fatty acid (FFA) release 197. Pharmacological inhibition or selective deletion of 5-HT_2B_ receptors in adipose tissues reduces HFD-induced AT lipolysis, lowers the subsequent release of FFA in the blood circulation, and improves insulin sensitivity and glucose tolerance 197,228. In addition, 5-HT_2B_ receptor activation leads to reduced energy expenditure in BAT by suppressing the uncoupling protein 1 (UCP1), which promotes energy storage and weight gain 229.

Together, these data suggest that elevated levels of peripheral 5-HT might facilitate the development of metabolic dysfunction and obesity. In line with this, patients treated with selective serotonin reuptake inhibitor (SSRI) antidepressants, medications that increase 5-HT availability by blocking its reuptake through the serotonin transporter (SERT), show reduced BAT thermogenesis 229, which could contribute to SSRI-induced weight gain and metabolic dysfunction, although some of these effects may occur independently of 5-HT signaling 230. Similarly, mice genetically deleted for the SERT (*Sert*^-/-^) exhibit metabolic dysfunctions such as glucose intolerance, insulin resistance, and obesity 231-233. Overall, these data support the involvement of 5-HT in the regulation of energy expenditure 234,235 and suggest that targeting peripheral 5-HT may constitute a therapeutic strategy for the treatment of obesity and associated metabolic dysfunctions.

### b. Diabetes

Diabetes mellitus refers to a group of metabolic disorders characterized by impaired glucose homeostasis. Type I (insulin-dependent) and Type II (insulin-resistant) diabetes are among the most clinically significant metabolic diseases, with Type II accounting for most cases 236. Accumulating evidence implicates both central and peripheral 5-HT signaling in the regulation of insulin signaling and glucose homeostasis, with dysregulations of 5-HT levels being associated with impaired insulin function and diabetes development 237.

Postprandial insulin secretion inhibits the breakdown of stored fat into FFA (lipolysis) and, instead, promotes the synthesis of fatty acids and their storage as triglycerides in fat cells (lipogenesis). Importantly, β-cells also express TPH1 (and TPH2) 238,239, produce 5-HT, and store 5-HT in the same vesicles as insulin 240. Upon glucose stimulation, 5-HT is co-released with insulin and acts in an autocrine and paracrine manner to potentiate insulin secretion-primarily via 5-HT_2B_ and 5-HT_3_ receptors on β-cells 27,241-243, and 5-HT_1F_ receptors on α-cells to suppress glucagon 240. In addition, 5-HT can directly promote insulin granule exocytosis via receptor-independent serotonylation 244, a post-translational modification mediated by transglutaminase 2 (TGM2), which covalently links 5-HT to target proteins such as Rab GTPases 245. This could alter cytoskeletal dynamics and intracellular signaling and enhance cellular motility and proliferation.

It is well established that patients with diabetes, both type I and II, exhibit elevated levels of plasma 5-HT, which correlate with their increased blood glucose levels 246-250. This elevation is accompanied by a decrease in platelet 5-HT storage 249 due to reduced basal uptake and enhanced spontaneous release of 5-HT 247,249. Rodent models such as streptozotocin-induced β-cell depletion mirror these findings, with elevated plasma 251 and gut 252,253 5-HT levels, increased density of enteric 5-HTergic neurons 254,255, and restoration of these changes following insulin treatment. In contrast, central 5-HT signaling is generally reduced in diabetic rodents, with decreased levels of 5-HT and TPH in the brainstem, lower density of 5-HT neurons, downregulated SERT, altered 5-HT_1A_ and 5-HT_2_ receptor expression 256-258, and diminished 5-HT release in the hypothalamus and cortex 257,259-most of which are also corrected by insulin therapy.

A key regulator of postprandial insulin secretion is GLP-1, an incretin hormone secreted by enteroendocrine L-cells in response to nutrient intake. GLP-1 acts on GLP-1 receptors (GLP-1R) expressed on pancreatic β-cells to enhance insulin release and suppress glucagon effects. GLP-1R is the target of widely used GLP-1 receptor agonists for the management of obesity, type 2 diabetes and, in the near future, MASLD 144. GLP-1 agonists also increase TPH expression in β-cells and stimulate 5-HT synthesis and its co-release with insulin 239. In the gut, GLP-1R is highly expressed in EC cells, where its activation robustly stimulates 5-HT secretion, establishing GLP-1 as an upstream regulator of 5-HT signaling 136. Conversely, 5-HT itself, through stimulation of 5-HT_4_ receptors on enteric neurons and L-cells, enhances GLP-1 release, forming a bidirectional feedforward loop 260​. Moreover, stimulation of 5-HT_4_ receptors improves intestinal barrier integrity and glucose tolerance in models of Type 1 diabetes 261 and HFD-induced metabolic dysfunction 262, respectively, paralleling GLP-1's protective effects 263.

GLP-2, co-secreted with GLP-1, complements its effects by maintaining mucosal integrity, promoting intestinal growth, and enhancing nutrient absorption through actions on GLP-2R, which are expressed on EC 5-HT cells and 5-HT-sensitive enteric neurons 264,265, further linking GLP-2 activity to 5-HTergic modulation of gut motility and epithelial function. GLP-2 thereby indirectly influences 5-HT availability and function, potentially enhancing both GLP-1 and 5-HT secretion. Meanwhile, 5-HT_4_ receptors, expressed on epithelial and neuronal compartments, mediate mucosal repair, anti-inflammatory effects, and promote peristalsis, functions that both overlap with and potentiate GLP-2's reparative actions.

This network of signaling reveals a tightly integrated endocrine and paracrine system wherein GLP-1, GLP-2, and 5-HT mutually regulate each other's release and downstream effects through synergistic receptor pathways. Disruption of any component can impair insulin secretion, contributing to the pathophysiology of diabetes. Collectively, these findings highlight a complex and bidirectional relationship between 5-HT and insulin in the regulation of glycemic levels and the pathophysiology of diabetes.

### c. Metabolic dysfunction-associated steatotic liver diseases

MASLD is the most common chronic liver disease worldwide, affecting 34-38% of adults in the global population. Associated with obesity, insulin resistance, and other features of metabolic syndrome, MASLD represents a growing public health challenge due to the potential progression of liver steatosis to steatohepatitis, fibrosis, cirrhosis, and hepatocellular carcinoma 266. MASLD is characterized by dysregulations of nutrient metabolism, notably lipids and carbohydrates, resulting from a combination of excessive dietary intake and impaired hepatic metabolic functions 76,144,267-273.

The presence of nutrients in the stomach stimulates 5-HT synthesis by TPH1 within the gastrointestinal tract, leading to elevated platelets levels of 5-HT in the bloodstream. The liver, being the first organ to receive blood from the gut via the portal vein, is exposed to these increased platelets 5-HT concentrations, at levels high enough to exert systemic, hormone-like effects on liver functions. Patients with obesity have increased basal TPH1 activity in the gut, resulting in a two-fold increase in plasma levels of 5-HT potentially reaching the liver 218. Consistently, in mice, genetic deletion of the serotonin transporter (SERT), which increases extracellular 5-HT levels, results in weight gain and liver steatosis 233.

Obesity associated with HFD induces liver steatosis and inflammation, increases platelet number and aggregation in the liver sinusoids 274, and increases TPH1 activity in the gut (duodenum, jejunum, and colon), which augments 5-HT release from the gut 222 and platelet-derived 5-HT to the hepatic portal vein 198. This elevation in TPH1 activity, as well as the subsequent elevation in 5-HT reaching liver, is likely implicated in the pathogenesis of metabolic liver diseases, as pharmacological inhibition of TPH1 reduces weight gain and adiposity, improves insulin sensitivity, and ameliorates liver steatosis in mice fed a HFD 199,219,224,225,275,276. Since several peripheral organs express TPH1 as mentioned above, the potential therapeutic effect of TPH1 inhibitors could thus result from combined effects on these organs.

Selective depletion of gut-derived 5-HT by genetic ablation of gut TPH1 (*villin*-cre: *tph1*^fl/fl^) improves HFD-induced liver steatosis and triglyceride accumulation, but does not affect other systemic energy metabolism features, such as body weight, plasma cholesterol and triglycerides, glucose tolerance, and adiposity, suggesting that gut-derived 5-HT is specifically involved in liver lipogenesis 198. This effect is likely mediated by 5-HT_2A_ receptors located on hepatocytes, as their selective ablation (5-HT_2A_ cKO, *Albumin-Cre*: *Htr2a*^fl/fl^) protects against HFD-induced steatosis independently of systemic energy homeostasis 198. Not only is steatosis diminished, but the inflammatory and fibrogenic responses are abolished in 5-HT_2A_-cKO mice upon HFD challenge, suggesting that 5-HT_2A_ antagonists could represent a treatment strategy for steatohepatitis and liver fibrosis 198,277,278. Similarly, pharmacologic blockade of 5-HT_4_ receptors (GR113808) prevents weight gain, insulin resistance, hepatic steatosis and inflammation induced by HFD in mice 279.

On the other hand, hepatocytes express 5-HT_2B_ receptors that do not seem to participate in liver steatosis development as no changes were observed in mice selectively ablated for 5-HT_2B_ receptors in hepatocytes (*Albumin-Cre*: *Htr2b*^fl/fl^) 198. Instead, hepatocytic 5-HT_2B_ receptors likely regulate glucose homeostasis by promoting gluconeogenesis through the stimulation of the two rate-limiting gluconeogenic enzymes: *fructose-1,6-bisphosphatase* and the *glucose-6-phosphatase,* and by inhibiting glucose uptake 197. In addition, 5-HT_2B_ receptors expressed in HSC, exert an inhibitory effect on liver regeneration by stimulating TGFβ expression, which also stimulates fibrogenesis 200. Interestingly, 5-HT_2B_ receptor expression in HSC is upregulated in fibrotic liver, suggesting its important role in the fibrogenesis process 200,280. In line with this, pharmacological inhibition or genetic deletion (*Htr2b^-/-^*) of 5-HT_2B_ receptors reduces liver fibrosis, promotes hepatocyte growth, and improves liver function in a mouse model of liver fibrosis (carbon tetrachloride (CCl4)-induced liver fibrosis mouse models) 200. Together, these data suggest that antagonizing 5-HT_2A_ receptors in hepatocytes and/or 5-HT_2B_ receptors in HSC could dampen the MASLD progression. A similar protection against HFD-induced hepatic steatosis was also observed in mice ablated for TPH1 or 5-HT_2B_ in adipocytes (*adiponectin-cre*; *Tph1^fl/fl^* or *Htr2b^fl/fl^*) or mice genetically deleted for the 5-HT_3a_ receptor (*Htr3a*^-/-^) 225,228.

5-HT could also exert its pathogenic effects indirectly, via 5-HT_2A_ receptor mediated upregulation of its degradation enzyme 281-284, the monoamine oxidase (MAO), located on mitochondria. Being the most exposed organ to gut-derived 5-HT, the liver is also the primary site of 5-HT metabolism with a high expressing of MAO 285. Higher 5-HT degradation may lead to overproduction of reactive oxygen species (ROS) and formation of free radicals, which could contribute to increased oxidative stress, causing lipid peroxidation, cellular damages, and liver inflammation 281-284. By stimulating the phosphorylation of c-Jun N-terminal kinase (JNK), p38 mitogen-activated protein kinase (p38 MAPK), signal transducer and activator of transcription 3 (STAT3) and NF-κB, the 5-HT degradation system can upregulate pro-apoptotic factors such as Bcl-2 Associated X-protein (Bax), cleaved-caspase 3 and cleaved-caspase 9, and downregulate anti-apoptotic factors like Bcl-2, which ultimately results in apoptosis, secretion of TNF-α and IL-1β, and the progression of liver diseases 281,282. Blocking 5-HT_2A_ receptors with the selective antagonist sarpogrelate has been shown to prevent MAO upregulation and reduce the resulting increase ROS byproducts, which further prevent the JNK/p38 MAPK/STAT3 cascade activation, and reduced inflammation and apoptosis, thereby preventing carbon tetrachloride (CCl4)-induced hepatotoxicity i*n vitro* and *in vivo* 281,282.

The progression of liver fibrosis is closely linked to both portal fibrosis and angiogenesis, two interconnected processes that result from the activation of portal myofibroblasts. These cells originate from various sources, including resident portal fibroblasts, perivascular mesenchymal cells, and potentially cholangiocytes undergoing transdifferentiation. In response to chronic injury and inflammation, they accumulate within the portal regions, where they drive extracellular matrix deposition and contribute to the formation of fibrotic septa that eventually bridge neighboring portal tracts. In the meantime, activated portal myofibroblasts release pro-angiogenic signals such as VEGF, PDGF, and Angiopoietin-2, promoting the growth of new capillary networks within the expanding fibrotic tissue. This close relationship between fibrogenesis and angiogenesis creates a self-reinforcing cycle that not only sustains the progression of fibrosis but also alters hepatic microcirculation by diverting blood flow and exacerbating portal hypertension, which may further amplify the fibrogenic response 286. 5-HT may not only modulate portal hypertension through the activity of 5-HT_1A_ and 5-HT_2A_ receptors 69,70,287,288, but could also regulate angiogenesis by modulating endothelial cells function through VEGF-dependent or -independent manners, involving various 5-HT receptors such as 5-HT_1B_, 5-HT_2A_, 5-HT_4_ or 5-HT_7_ 289-293.

Both alterations in blood and bile flow can also promote liver disease, notably through the development of cholestasis, a condition characterized by the intrahepatic accumulation of bile acids, which can induce hepatocellular injury, inflammation and fibrosis. In response to sustained biliary stress, the liver undergoes bile duct remodeling, characterized by ductular reaction, proliferation of cholangiocytes (the epithelial cells lining the bile duct), and periductal fibrosis, which further perpetuate portal inflammation and fibrosis. Cholangiocytes express both TPH1 and TPH2, enabling local production of 5-HT 25,26. This locally produced 5-HT acts in an autocrine fashion to inhibit cholangiocyte proliferation via the activation of 5-HT_1A_ receptors 26. In rodent models of cholestasis induced by bile duct ligation (BDL), TPH1 & 2 expression is upregulated while MAO expression is downregulated, leading to increased intra-biliary 5-HT level 26. Genetic ablation of TPH2 results in an ~80% reduction in biliary 5-HT, promoting excessive proliferation of cholangiocytes and hepatocytes, expansion of immature ductules, and increased recruitment of liver progenitor cells. This aberrant proliferative response promotes extensive ductular remodeling and exacerbates liver fibrosis 26. 5-HT secreted by cholangiocytes also acts in a paracrine manner on HSC to stimulate their trans differentiation into myofibroblasts and augment TGF-β1 secretion. In turn, TGF-β1 suppresses TPH2 expression in cholangiocytes, relieving the inhibitory effect of 5-HT on ductular proliferation and reinforcing a feedforward loop that perpetuates both fibrogenesis and ductular expansion 26. BDL also upregulates 5-HT_2A, 2B, 2C_ receptors expression in both cholangiocytes and HSC 25. Stimulation of these receptors by 5-HT further enhances bile duct mass, collagen deposition, proinflammatory cytokine production, and secretion of senescence-associated secretory phenotypes (SASPs), whereas their antagonism significantly reduces these responses and attenuates liver fibrosis 25.

Together, these findings highlight the complex role of 5-HT signaling in regulating hepatic metabolism, inflammation, cholangiocyte proliferation, biliary remodeling, and fibrogenesis. Through distinct receptor pathways and tissue-specific effects, 5-HT therefore acts as a pivotal modulator in the progression of MASLD (and cholestatic liver diseases).

### d. Alcohol associated liver diseases

While the role of central 5-HT signaling in alcohol use disorders and associated mood dysregulations have been extensively investigated, showing that chronic alcohol consumption enhances brain 5-HT innervation and signaling, with 5-HT_1A_ receptors playing a key role in binge-alcohol drinking behavior 294-301, little is known regarding the potential role of peripheral 5-HT signaling in ALD.

Nevertheless, elevated blood and urine levels of 5-HT have been reported in patients with severe alcohol-associated cirrhosis and in corresponding animal models 302-304, likely resulting from elevated expression of intestinal TPH1 304 and reduced storage of 5-HT in blood platelets 305-307. Notably, mice genetically deleted for intestinal TPH1 are protected against alcohol-induced liver steatosis, inflammation, and alterations in lipogenesis pathways, probably by preventing neutrophil mobilization into the liver 308 and nuclear translocation of Sterol Regulatory Element-Binding Protein 1 (SREBP1) 304. Similarly, hepatocyte-specific knockout of 5-HT_2A_ receptors confers protection against alcohol-associated steatosis, and SREBP1 activation, thereby attenuating liver endoplasmic reticulum stress and inflammation 304. Together, these findings suggest that intestinal 5-HT, acting through 5-HT_2A_ receptors, contributes, at least in part, to the early stages of ALD. However, further studies are warranted to clarify the respective role of intestinal and liver-secreted 5-HT in the progression towards more advanced stages of the liver disease.

### e. Hepatocellular carcinoma

5-HT likely exerts a potent pro-tumorigenic factor in HCC, influencing both disease progression and prognosis through multiple intertwined mechanisms. Elevated systemic and intraplatelet levels of 5-HT have been consistently reported in patients with HCC compared to cirrhotic controls, with plasma 5-HT concentrations emerging as a potentially sensitive and non-invasive biomarker for early-stage HCC detection across various underlying etiologies 8,309,310. Moreover, elevated levels of 5-HT storage in platelets have been associated with increased risk of tumor recurrence, early relapse, and poor overall survival, suggesting that platelet-derived 5-HT acts as both a systemic marker and a local driver of tumorigenesis 311-316. Mechanistically, 5-HT promotes hepatic cancer cell proliferation, invasion, and metastasis primarily via receptor-mediated signaling, notably through the 5-HT_2B_ receptor, which is highly upregulated in HCC tissues 317-319. Activation of 5-HT_2B_ by 5-HT enhances the expression and nuclear activity of YAP (Yes-associated protein), a central effector of the Hippo pathway, thereby driving tumor growth through downstream ERK and YAP phosphorylation 319. Interestingly, high levels of both intraplatelet 5-HT and YAP are independently associated with poor clinical outcomes, reinforcing the functional significance of the 5-HT-YAP axis in HCC pathogenesis 315,316.

Beyond 5-HT_2B_, HCC tissues show a distinct remodeling of 5-HT receptor expression. Upregulation of 5-HT_1B_, 5-HT_1D_, and 5-HT_7_ receptors, and downregulation of 5-HT_2A_ and 5-HT_5_ receptors have also been observed in tumors 317,318. Antagonism at 5-HT_7_ receptors significantly reduces tumor size *in vivo*, suggesting a proliferative or survival role of this receptor in liver malignancy 318. 5-HT_1B_ and 5-HT_1D_, similarly overexpressed in HCC, have been involved in the regulation of epithelial-mesenchymal transition and cell invasiveness in pancreatic cancer, pointing to a potential role in HCC progression, yet their exact contribution remains unclear in liver tissue.

5-HT also exerts tumor-promoting effects independently of receptor activation, through serotonylation. In HCC, elevated TGM2 expression correlates with poor differentiation, high levels of the HCC marker alpha-fetoprotein (AFP), and advanced tumor stage, with pharmacological inhibition of serotonylation suppressing tumor growth 321. These findings suggest that serotonylation may contribute to tumor aggressiveness in HCC.

Collectively, these studies depict 5-HT as a potential modulator of HCC, acting through platelet-derived paracrine mechanisms, receptor-specific signaling, and epigenetic modifications. This positions 5-HT not only as a biomarker for early detection and prognosis but also as a promising target for therapeutic intervention in hepatocellular carcinoma.

### f. Cardiovascular diseases

Cardiovascular diseases (CVD) represent major comorbidities of metabolic dysfunction and chronic liver disorders such as MASLD and HCC, reflecting their convergence within a shared cardiometabolic continuum. Beyond its hepatic and metabolic roles, 5-HT functions as a potent vasoactive mediator that regulates vascular tone, endothelial function, platelet aggregation, and myocardial remodeling. More than 95 % of circulating 5-HT is synthesized peripherally by enterochromaffin cells and stored in platelets, from which it is released upon activation during thrombus formation or acute inflammation 322. Physiologically, 5-HT contributes to vascular homeostasis through the balanced activation of vasoconstrictive 5-HT_2A/2B_ and vasodilatory 5-HT_1B/7_ receptors 322,323. Under metabolic stress, hyperlipidemia and insulin resistance disrupt this equilibrium, promoting endothelial dysfunction, oxidative stress, and thrombosis 237,324.

Clinical and experimental data demonstrate that platelet-derived 5-HT contributes to atherogenesis, vascular inflammation, and myocardial injury. Elevated plasma 5-HT and reduced platelet storage are reported in obesity, diabetes, and MASLD, correlating with vascular stiffness, carotid intima-media thickness, and coronary artery calcification 325,326. During coronary artery disease, platelet activation within the ischemic microenvironment triggers massive 5-HT release, which acts as a chemoattractant and activator of neutrophils 308. In TPH1^⁻/⁻^ mice or following chronic SSRI treatment, depletion of platelet-derived 5-HT markedly reduces neutrophil recruitment, degranulation, and myocardial injury 308,327,328. Mechanistically, 5-HT signaling through 5-HT7 receptors on neutrophils increases intracellular calcium, promoting CD11b externalization, myeloperoxidase (MPO) release, and reactive oxygen species (ROS) production, which enhance neutrophil adhesion to platelets and injured endothelium, aggravating reperfusion injury 308,328. Conversely, pharmacological depletion of platelet 5-HT or long-term SSRI administration mitigates this inflammatory cascade and confers protection against ischemia/reperfusion injury 329. In humans, plasma 5-HT levels correlate positively with neutrophil CD11b and MPO expression in acute coronary syndrome, whereas SSRI therapy suppresses both markers 328. Consistently, patients treated with SSRIs display a reduced risk of first myocardial infarction, consistent with decreased platelet 5-HT uptake and aggregation 330. At the cardiac level, 5-HT_2B_ receptor activation in cardiomyocytes and fibroblasts triggers ERK1/2- and TGF-β-dependent remodeling, leading to hypertrophy and diastolic dysfunction 331. Notably, similar 5-HT_2B_-driven fibrogenic programs operate in hepatic stellate cells during fibrosis 200, suggesting shared serotonergic mechanisms underpinning cardiac and hepatic remodeling. Elevated circulating and hepatic 5-HT levels observed in cirrhosis and HCC correlate with systemic and portal hypertension 303,332-334, further linking hepatic 5-HT dysregulation to extrahepatic vascular pathology. Recent findings also implicate intestinal tryptophan (Trp) metabolism in the regulation of systemic 5-HT levels and cardiovascular risk. Hence, dietary Trp is catabolized via three main pathways: (1) the kynurenine (Kyn) pathway in intestinal epithelial cells through indoleamine 2,3-dioxygenase 1 (IDO1) or in the liver via Trp 2,3-dioxygenase (TDO); (2) the microbial indole pathway, which converts Trp into indole metabolites; and (3) the 5-HT synthesis pathway EC cells via the TPH1 329. In mice lacking intestinal IDO1 or deprived of dietary Trp, the Kyn pathway blockade redirects metabolism toward 5-HT synthesis, elevating gut-derived and circulating 5-HT, compromising barrier integrity, and promoting systemic inflammation and atherosclerotic plaque formation 329. Pharmacological inhibition of TPH1 normalizes these effects by reducing gut inflammation and plaque burden, whereas exogenous 5-HT supplementation aggravates vascular lesions 329. At the cellular level, these deleterious effects are orchestrated through endothelial 5-HT_1B/2A/2B_ receptors, which regulate vascular tone and inflammatory signaling, and macrophage 5-HT_2A/2B/7_ receptors, which drive cytokine release, inflammasome activation, and leukocyte recruitment during atherogenesis and ischemic injury 292,335-338.

Collectively, these findings identify 5-HT as a unifying molecular link between metabolic, hepatic, and cardiovascular disorders. Dysregulated peripheral 5-HT not only promotes hepatic steatosis, fibrosis, and tumor progression but also drives vascular inflammation and myocardial remodeling, amplifying morbidity and mortality across the metabolic syndrome. Understanding 5-HT signaling within this integrated liver-gut-cardiovascular axis may thus reveal therapeutic opportunities capable of concurrently mitigating hepatic and cardiovascular complications.

## IV. Conclusion and Clinical Considerations

Extensive evidence suggests that 5-HT exerts context-dependent effects on metabolic and liver health, mediated through both central and peripheral pathways. Elevated peripheral 5-HT levels are generally associated with deleterious outcomes, promoting hepatic steatosis, inflammation, fibrogenesis, and tumor progression, thereby contributing to the development of MASLD, ALD, and HCC. In contrast, physiological 5-HT signaling can exert beneficial actions by stimulating hepatocyte proliferation and liver regeneration, particularly following injury or hepatectomy, creating a therapeutic paradox. These opposing outcomes likely depend on the relative contribution of gut-derived vs locally synthesized 5-HT, each exerting organ- or tissue-specific effects according to the repertoire of 5-HT receptor subtypes expressed. At the central levels, 5-HT primarily modulates appetite, energy expenditure, and autonomic outflow, indirectly influencing liver metabolism and systemic homeostasis*.* Although 5-HT cannot cross the blood-brain barrier, evidence suggests that peripheral 5-HT levels may inversely influence central 5-HT synthesis, notably by modulating Trp availability.

This duality highlights the importance of integrating both central and peripheral 5-HT signaling when considering therapeutic interventions. Decreasing overall 5-HT levels using the non-selective inhibitor of both central and peripheral TPH (PCPA), was shown to reduce obesity and adiposity, as well as diet- and alcohol-induced hepatic steatosis and inflammation 198,219,224,225,304,339. However, prolonged brain 5-HT depletion by PCPA is also known to produce anxiety 340, to impair motivation 341 and cognitive functions 342, and to increase aggressivity 343 and vulnerability to stress 344. While reducing overall 5-HT levels may seem like a feasible approach to halting the progression of liver disease, it could also greatly affect emotions and mood. Resolving this contradiction is essential to devise new 5-HT-targeting therapeutics for the treatment of chronic liver disease. In line with this, novel TPH1 inhibitors that do not cross the blood brain barrier have been developed, revealing that inhibition of peripheral 5-HT synthesis is sufficient to protect against diet-induced obesity, reduce blood glucose levels, adiposity, and prevent diet-induced liver steatosis 199,275.

Conversely, this suggests that drugs elevating 5-HT levels, such as selective serotonin reuptake inhibitors (SSRIs) or MAO inhibitors (MAOI) antidepressants may aggravate liver steatosis and steatohepatitis. For instance, 5-HTergic medications, particularly SSRIs, have been consistently associated with adverse metabolic and hepatic effects 345-350. Chronic exposure to SSRIs such as sertraline, fluoxetine, and citalopram induces hepatotoxicity by impairing the liver's ability to metabolize drugs and fatty acids 351,352. In epidemiological studies, antidepressants with high affinity for the serotonin transporter have been linked to elevated serum LDL cholesterol levels 353, while clinical trials in anxiety or depressive disorders reveal divergent effects across molecules. For instance, fluoxetine was associated with reductions in body weight and lipid markers, whereas paroxetine, citalopram, and sertraline were linked to increased weight, waist circumference, and systemic glucose, LDL, and triglycerides 354. Long-term use of SSRIs or tricyclics has further been correlated with an increased risk of diabetes mellitus 355, and several reports confirm that fluoxetine, sertraline, venlafaxine, and related agents unfavorably impact lipid profiles, with venlafaxine showing the strongest association with dyslipidemia 356,357. Fluoxetine has particularly been implicated in hepatic lipid accumulation via a dual mechanism, upregulating the SREBP1c-ACC1-FAS lipogenic axis through p38 MAPK signaling while suppressing lipolysis via downregulation of CES1/3 358. This is compounded by fluoxetine-induced increase in hepatic 5-HT synthesis, which in turn promotes steatosis, an effect reversed by TPH inhibition 359. Human and murine studies further confirm that fluoxetine elevates serum triglycerides and LDL and increases liver expression of lipogenic genes while suppressing adipogenic and β-oxidation markers 360,361. Similar hepatotoxic effects, such as acute liver injury, cholestasis, vanishing bile duct syndrome, chronic fibrosis, Kupffer cell hyperplasia, glycogen depletion, and nuclear damage have been reported for sertraline 362-364. Similarly, MAO isoforms A and B are critical players in the intersection between 5-HT and liver diseases. MAO-A is upregulated in MASH liver and mediates oxidative stress-induced hepatocellular damage 365. Similar findings in patients with obesity show elevated MAO-A expression correlating with increased ROS and vascular dysfunctions 366, while MAO-B levels are markedly increased in fibrotic liver cells 367. Blockade of MAO activity using inhibitors (e.g., clorgyline, pargyline) has shown beneficial effects on food intake and obesity markers in mice 368, suggesting that selective modulation of MAO may offer therapeutic avenues.

Beyond antidepressants, 5-HT receptor agonists such as triptans are also associated with hepatotoxicity. Sumatriptan and rizatriptan, 5-HT_1D_ receptors agonists, have been shown to cause mitochondrial and lysosomal dysfunctions in hepatocytes, marked by lipid peroxidation and oxidative stress 369. Yet not all serotonergic drugs are deleterious. Buspirone, a 5-HT_1A_ receptor agonist, reduces oxidative stress and protects against CCl₄-induced liver fibrosis 370, and portal vein-targeted 5-HT_1A_ modulation has shown promise in reducing portal hypertension 288. Several 5-HT receptors such as 5-HT_7_, 5-HT_2B_ and 5-HT_1D_ have also been involved in the deleterious effects of 5-HT on HCC progression *in vitro*. For instance, 5-HT increases the proliferation of HCC cells by reducing β-catenin degradation and subsequently enhancing Wnt/β-catenin signaling pathway, a process attenuated by a 5-HT_7_ antagonist 318. Pharmacological inhibition of 5-HT_2B_ receptors also reduces the proliferation and invasiveness of HCC cells by attenuating the activation of mammalian target of rapamycin (mTOR) and YAP pathways 319. Finally, genetic silencing of 5-HT_1D_ receptors was shown to inhibit the proliferation, migration and invasion of HCC cells, by attenuation Pi3K/Akt-dependent epithelial-mesenchymal transition (EMT) 371. Interestingly, emerging evidence suggests a paradoxical protective role of SSRI and tricyclic antidepressants in HCC. By inducing cancer cell autophagy, inhibiting tumor growth, and synergizing with anti-cancer agents, such as sorafenib, SSRIs likely decrease mortality in HCC patients 372-379. Furthermore, by reducing adiposity, hepatic steatosis, hepatocyte ballooning and liver fibrosis induced by HFD- or choline-deficient HFD, 5-HT_2A_ receptor antagonists have recently emerged as promising therapeutic candidates for MASH, MASLD and HCC 277,380.

Taken together, these observations highlight the complex and ambivalent role of 5-HT signaling in liver health. While some 5-HT targeting therapeutics show potential for modulating metabolic, inflammatory, or neoplastic processes within the liver, the unintended consequences of chronic 5-HTergic modulation-particularly through SSRIs and MAOI-raise significant safety concerns. These include disruptions in lipid and glucose homeostasis, exacerbation of steatosis, hepatocellular injury, and fibrosis. The liver's central role in drug metabolism renders it especially vulnerable to sustained 5-HT input, whether via enhanced serotonin levels, altered degradation pathways, or receptor overstimulation.

These findings highlight the temporal and receptor-specific duality of 5-HT signaling in chronic liver disease. Although 5-HT contributes to fibrogenesis and cirrhosis development, it may also participate in limiting tumor aggressiveness or supporting hepatic regeneration under specific conditions. Therapeutic strategies aiming to modulate serotonergic pathways in chronic liver disease must therefore consider both the disease stage and the cellular targets involved in 5-HT signaling. As such, repositioning serotonergic drugs for the treatment of chronic liver diseases demands a highly cautious, mechanism-guided approach. It will be essential to discriminate between receptor-specific effects, distinguish central versus peripheral actions, and identify patient profiles most likely to benefit from such interventions.

## Figures and Tables

**Table 1:** List of hepatic cytochromes regulated by central 5-HT signaling, their function, and the links to liver physiology, metabolism dysregulation, and liver diseases.

Name	Functions	Link with chronic liver diseases	Ref
CYP1A1	-generation of ROS -cholesterol, arachidonic acid and glucose metabolism -immune response	-lipid deposition, cholesterol accumulation, fatty liver -steatohepatitis, neutrophil infiltration -proliferation and differentiation of HSC -fibrosis -HCC	381-385
CYP1A2	-generation of ROS -cholesterol metabolism and lipid peroxidation	-metabolic dysfunction -neutrophil infiltration -cirrhosis -HCC	383,386
CYP1B	-steroid hormone -lipid, glucose, and vitamin D metabolism -fat synthesis	- insulin sensitivity	387
CYP2A	-steroid metabolism	-hepatitis -cirrhosis	388
CYP2C11	-steroid hormone, vitamin D, antidepressants, and antipsychotics metabolism	- liver injury - alcohol-related liver disease - fibrosis	389-391
CYP2D6	-immune functions -promotes 5-HT synthesis	-autoimmune liver diseases	392
CYP3A	-bile acid metabolism -inflammatory cytokine release	-fibrosis -cirrhosis	393
